# A Dataset to Study Pragmatic Language and Its Underlying Cognitive Processes

**DOI:** 10.3389/fnhum.2021.666210

**Published:** 2021-06-17

**Authors:** Jalil Rasgado-Toledo, Fernando Lizcano-Cortés, Víctor Enrique Olalde-Mathieu, Giovanna Licea-Haquet, Miguel Angel Zamora-Ursulo, Magda Giordano, Azalea Reyes-Aguilar

**Affiliations:** ^1^Department of Behavioral and Cognitive Neurobiology, Instituto de Neurobiología, Universidad Nacional Autónoma de México, Juriquilla, Mexico; ^2^Department of Psychobiology and Neuroscience, Facultad de Psicología, Universidad Nacional Autónoma de México, Mexico City, Mexico

**Keywords:** resting state fMRI, language-task fMRI, pragmatic language, functional connectivity, cortical thickness

## Introduction

Pragmatics studies the social-cognitive basis of communication that is crucial to the understanding of the non-literal meaning of an expression. This includes speech acts, metaphors, proverbs, idioms, and irony (Gibbs, 2002; Scott-Phillips, 2017). Pragmatic comprehension requires cognitive functions such as attention, the ability to use and store information (i.e., memory), comprehension of the structures of utterances (i.e., grammatical rules), integration of information from different sources (i.e., beliefs about the speaker and situation), empathy, the understanding of the mental states of others [i.e., theory of mind (ToM)], identification of speech properties, such as speed, intonation, gestures, and facial expression (i.e., paralinguistics), all guided by context (Van Dijk, 1977; Loukusa and Moilanen, 2009). Several psychiatric and neurological disorders like autism, Parkinson's disease, and schizophrenia share a deficit in pragmatic comprehension, besides the impairments in executive function or ToM, leading to the hypothesis that the latter are involved in pragmatic comprehension (Stemmer, 2017).

Pragmatics allows to understand the intended meaning of another speaker, which has been measured in a variety of ways: jokes (Zajdman, 1995; Canal et al., 2019), ironic stories (Monetta et al., 2009; Rivière et al., 2018; Zajaczkowska and Abbot-Smith, 2020), indirect speech acts (Searle, 1975; Domaneschi et al., 2017; Licea-Haquet et al., 2019), and taking turns tasks (Levinson, 2016; Seuren et al., 2021). Moreover, some tests attempt an in-depth evaluation, such as the “Pragmatic Language Skills Inventory (PLSI)” (Gilliam and Miller, 2006), the “Pragmatic Language Test” (Phelps-Terasaki and Phelps-Gunn, 2007) “The Listening Skills Test” (Lloyd et al., 1995), and the “Understanding Ambiguity Test” (Rinaldi, 1996), with a particular focus on some aspects of pragmatics. Likewise, the “Strange Stories Test” (Happé, 1994) measures pragmatic performance and other skills such as ToM. The evaluation of pragmatic capacities is very broad, and it usually includes the assessment of underlying cognitive processes that are needed for understanding the intended meaning according to the context.

The cognitive processes believed to participate in pragmatic language have been associated with specific brain structures and functional networks. Thus, the inferior frontal gyrus, the middle frontal gyrus, and the superior temporal gyrus are known as the core of the left perisylvian language network of the brain for phonological, syntactic, and semantic knowledge (Hagoort, 2017). However, pragmatic language processing goes beyond this left neural network, including a bilateral frontotemporal and medial prefrontal network, which is engaged by pragmatic form and stimulus configuration, establishing the “pragmatic language network (PLN)” (Reyes-Aguilar et al., 2018).

Recently, studies have evaluated some aspects of pragmatic comprehension through novel paradigms and new analytical approaches to decipher the neural correlates of pragmatic inferences. For example, some studies have questioned whether pragmatic understanding is an independent module or is a submodule of ToM (Bosco et al., 2018). The study by Powell et al. (2019) suggests that pragmatic intent does not recruit areas related to ToM and that it relies more on self-referential memory. In this study, ToM-related regions were recruited when meaning was recovered in the context of ambiguity. On the contrary, the study by Feng et al. (2021) suggests that ToM-related regions are recruited by indirect replies and that this activation is modulated by the level of contextual relevance. Other authors have proposed a dynamic intention processing network (IPN) (Enrici and Adenzato, 2019) that partially overlaps with the regions of ToM. Their proposal is based on the results of various neuroimaging studies in healthy subjects, in which they showed that this network is differentially activated according to the nature of the intention being processed, for example, if it is a private vs. a communicative intention. A different approach for studying brain networks that support cognitive functions, e.g., language and their lateralization, is the analysis of functional brain connectivity in a resting state. For instance, Zhang et al. (2021) have explored the status of the language network using this approach. Their results suggest that resting-state data could be an indicator of language abilities and a potential biomarker for studying the association between age and cognition.

This topic has been the main line of research in our laboratory, where the principal objective is to understand how we use language, in particular, its pragmatic components, including non-literal communication. With this purpose in mind, we designed various paradigms to evaluate the neural correlates of comprehension of specific pragmatic forms. We used neuroimaging techniques, i.e., MRI, and a battery of psychometric tests to evaluate the association between pragmatic comprehension and cognitive functions, including executive functions and ToM. Recently, we conducted an analysis with some of the fMRI language task data included in this study, in which we detected increased activation and functional connectivity in regions of the left neural perisylvian network and motor regions, such as the precentral gyrus, the supplementary motor area (SMA), and the cerebellum. We focused on these regions for the evaluation of asymmetry and homotopy related to some components of language. In particular, we wanted to explore the functional connectivity asymmetry in relation to manual preference as a measure indirectly related to language and verbal fluency as an indication of verbal ability (Hervé et al., 2006; Mazoyer et al., 2014).

## Methods

### Data Acquisition

All brain MRI data were acquired in different schedules over 4 years (2016–2019) in a variety of different pragmatic language study protocols. Participants in each of the six projects were scanned using a 3.0 T General Electric Co., Boston MA, Discovery-MR750 using a 32-channel head coil. Descriptions for each project are described in Supplementary Material.

Every protocol included high-resolution structural 3D-T1-weighted images with spoiled gradient recalled (SPGR), voxel size = 1 mm x 1 mm x 1 mm, flip angle = 12°, slice thickness = 1, repetition time (TR) = 8.1 ms, echo time (TE) = 3.2 ms, inversion time = 0.45, and field of view = 256 × 256 mm, covering the whole brain.

Five projects had functional task images while four had functional resting-state data. Both type of functional images were acquired using a T2*-weighted echo planar (EPI) sequence, using a 32-channel head coil, flip angle = 90°, 38 slices, slice thickness = 4, TR = 2000 ms, TE = 40 ms, a 64 × 64 matrix and final voxel size = 4 × 4 × 4 mm isometric voxel, field of view = 256 × 256 mm.

### Psychometric Data

The behavioral data obtained varied between protocols; however, all subjects share some psychometric tests. Of these, we present mean, SD, and ranges: Verbal Fluency, 22.63 (SD = 6.84; range 7–40); laterality coefficient by Edinburgh Handedness Inventory (EHI) [(Oldfield, 1971)], 74.86 (SD = 31.72; range −100–100); and the Short Story Task Comprehension scores, 6.84 (SD = 2.04; range 0–10). These data are provided on the OpenNeuro repository.

### Participants

The sample includes 145 neurotypical volunteers, Mexican-born participants (79 females and 66 males) with Spanish as their native language, aged 17–35 years (median 23.46), with a range of 12–22 years of education (median 15.7). No psychological distress or psychiatric disorders were detected by the Spanish version of Symptom Checklist 90 (SCL-90, mean 0.63, SD 0.46). Participants showed normal verbal comprehension measured by the Wechsler Adult Intelligence Scale (WAIS) (mean 105.2, SD 13.2), and no structural brain abnormalities were observed by a visual inspection of all structural images. Participants were asked to perform behavioral tasks of language comprehension, ToM, and executive functions to characterize cognitive pragmatic skills. Psychometrics and fMRI tasks were applied in Spanish. All participants were informed of study procedures and signed an informed consent form for each protocol approved by the internal Committee on Ethics, which also approved the experimental protocol, in compliance with the federal guidelines of the Mexican Department of Health (http://www.salud.gob.mx/unidades/cdi/nom/compi/rlgsmis.html), which agree with international regulations. Participants were recruited through announcements in nearby universities, places of interest, and by word of mouth.

### Format Description

All data are formatted using the Brain Imaging Data Structure (BIDS), which is an organization and descriptor of MRI datasets to unify the majority of projects in the field. The organization follows a specific pattern name related to acquisition modality in Neuroimaging Informatics Technology Initiative (NifTi) format with data descriptions represented by a JavaScript Object Notation (JSON) for MRI metadata and tab-separated value (TSV; .tsv) for task stimulus presentation time (Gorgolewski et al., 2016). For further details, please refer to the corresponding documentation (bids.neuroimaging.io/).

### MRI Quality Control

An MRI quality control of the dataset was run using MRIQC, a tool for extracting quality measures used to exclude problematic acquisitions through analysis of modularity, integrability, interoperability, noise, and artifacts measures, and spatial and temporal information, among other metrics (Esteban et al., 2017). Exported as.html and.json reports, they can be checked in the dataset. For further details, please refer to the corresponding documentation (mriqc.readthedocs.io/).

### Experimental fMRI Tasks

Five functional tasks on the present dataset are described in Supplementary Material. In the OpenNeuro repository (https://openneuro.org/datasets/ds003481/versions/1.0.2), we also provided a brief description of each project (e.g., description of the sample, procedure, and stimuli) in.json files and the order of stimulus presentation for the fMRI tasks.

### Pre-processing

For cortical thickness, image and statistical analyses were performed using FreeSurfer v.5.3 (http://surfer.nmr.mgh.harvard.edu/; Dale et al., 1999) as part of fMRIprep v1.1.4 (Esteban et al., 2019) preprocessing (https://fmriprep.readthedocs.io/). Cortical thickness values were calculated with aparcstats2table FreeSurfer function, parcellated with the Desikan-Killiany Atlas (Desikan et al., 2006). For asymmetry and homotopy analyses, we used resting preprocessing files (105 subjects) from fMRIprep and registered them to MNI152Lin template.

## Structural Connectivity Analysis

### Cortical Thickness as a Psychometric Predictor

**Figure 1:**
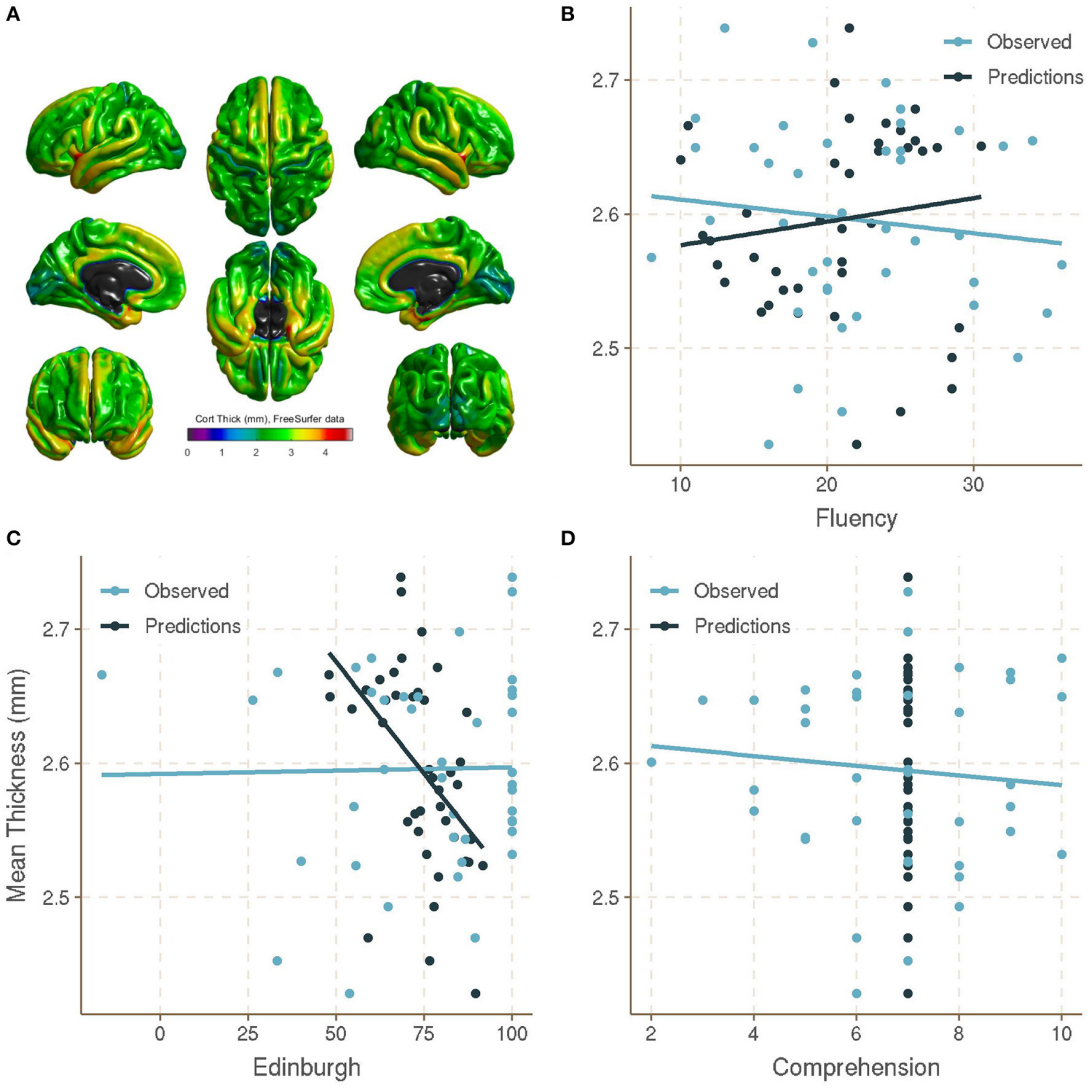
**(A)** Mean cortical thickness extracted using FreeSurfer processing and parcellated with Desikan-Killiany Atlas. **(B)** Scatterplots of observed Verbal Fluency values (x axis) and mean cortical thickness (mm) (y axis) and K-nearest neighbor (KNN)-predicted values for Verbal Fluency, **(C)** Edinburgh Handedness Inventory (EHI) coefficients, and **(D)** Short-Story Task (SST) comprehension scores.

Using a k-nearest neighbors (KNN) machine learning approach, we tested the reliability to predict the Edinburgh laterality coefficient, verbal fluency, and reading comprehension scores from cortical thickness measures (Figure 1A). For this purpose, we calculated the Euclidean distance of each cortical thickness region in search of similarities to create the regression model and identify the most influential regions as the most critical variables for the model adjustment, described as a percentage (threshold > 75).

For the verbal fluency task, with an automatic adjustment of two neighbors (k), differences between KNN-predicted and observed values were not so large, with a root mean square error (RMSE) of 9.08 (Figure 1B). The regions that most influenced the model adjustment for predicted values were right inferior frontal–pars opercularis = 100.00, left entorhinal cortex = 84.58, right rostral anterior cingulate = 79.64, and left lingual gyrus = 76.05. In general, some predicted values were close to the observed values in the test, suggesting cortical thickness as a possible descriptor of verbal fluency. However, for the Edinburgh laterality coefficient, KNN-predicted values were poorly calculated, with a high RMSE (26.72; *k* = 6), which is reflected in the poor performance of the model in calculating possible scores according to cortical thickness (Figure 1C). Regions that influenced this model were right inferior frontal gyrus–pars opercularis = 100.00, right parahippocampal gyrus = 87.05, right inferior temporal gyrus = 85.38, right inferior frontal-pars triangularis = 85.25, pars orbitalis = 83.72, and left middle temporal gyrus = 79.15. Most of these regions are directly involved with asymmetrical functions, such as language and visuospatial processing, indirectly or weakly with handedness (Hervé et al., 2006; Kong et al., 2018). Finally, for Reading Comprehension Subscale (SST), predicted values were poorly calculated, being mostly seven, despite the high range of scores on the test (RMSE = 1.98, *k* = 70), and the most relevant regions for the model were right precuneus = 100.00, right medial orbitofrontal cortex = 87.95, right supramarginal gyrus = 77.06, and left inferior parietal gyrus = 76.68 (Figure 1D).

### Asymmetry and Homotopy Analyses of Resting State

To test the status of the resting state and the association between hemispheric connectivity and psychometric scores, we calculated an asymmetry index as described in the study of Gracia-Tabuenca et al. (2018). This index describes the normalized difference of intra-hemispheric weighted degree of asymmetry for each pair of mirror regions of interest (ROIs) within a symmetric atlas [AAL3 (Rolls et al., 2020)], where a positive value indicates a higher degree of asymmetry in the right hemisphere while a negative one means higher asymmetry in the left.

In addition, we calculated the functional connectivity between each pair of mirror ROIs within the AAL3, known as homotopic connectivity (Zuo et al., 2010; Gracia-Tabuenca et al., 2018). The analysis was performed on areas that have been related to language and motor processing: frontal inferior gyrus triangularis, superior temporal gyrus, precentral gyrus, SMA, and cerebellum: crus I, II, and lobe 10 (Kellermann et al., 2012; D'Mello et al., 2017).

**Figure 2:**
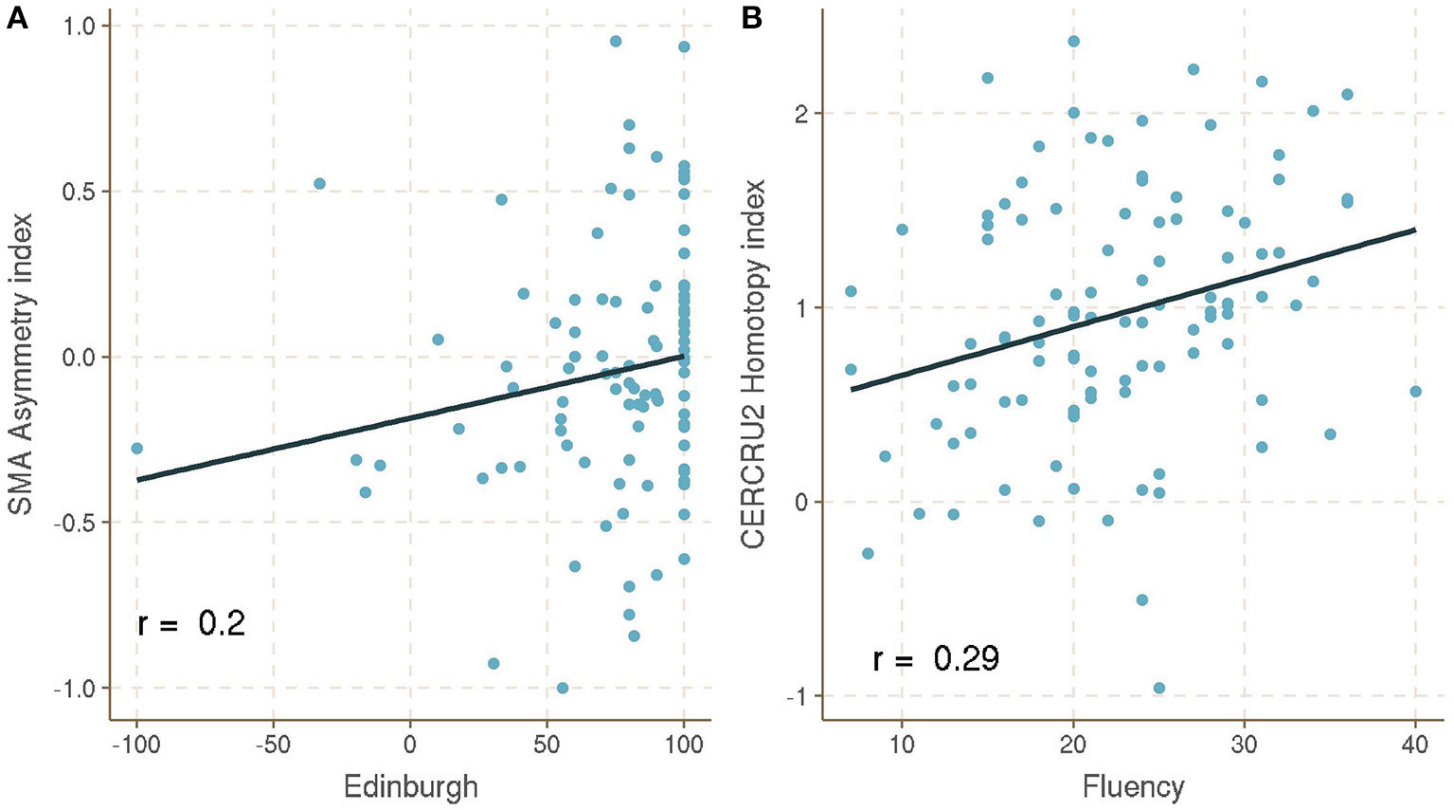
**(A)** Asymmetry index and **(B)** homotopy functional connectivity between Verbal Fluency Test and EHI with significant brain regions, false discovery rate (FDR) corrected, calculated using Spearman's rank correlation. SMA, supplementary motor area; CERCRU2, crus II of cerebellar hemisphere.

For the asymmetry index (Figure 2A), the only significant value was the score in the EHI with SMA (*r* = 0.204, *p* < 0.041), a common motor region implicated in asymmetric motor skills and with differential activations between hemispheres (Scholz et al., 2000; Dinomais et al., 2016).

Homotopic connectivity showed different results (Figure 2B). Specifically, verbal fluency was associated with cerebellar hemisphere crus II [*r* = 0.291, *p* < 0.025, false discovery rate (FDR) corrected], which is functionally connected with the cortical language network, and has been suggested to support semantic prediction in speech production and comprehension (Kellermann et al., 2012; D'Mello et al., 2017).

## Recommended Uses

The Github link includes all the necessary scripts and files to replicate the analyses presented here. We also provide .mriqc files and the quality-checked .json in the dataset to rate the quality of each sequence. We also indicate the best sequences according to our ratings. We acquired images from a total of six project cohorts described in Supplementary Material. The common sequence was high-resolution T1-weighted imaging, which may be used to assess brain volume (white and gray matter), cortical thickness, or surface area and to explore correlations with psychometric measures using graph theory (GT) approaches. A second major sequence was resting functional imaging, which may be used for evaluating the connectivity status using seed-based or voxel-wise connectivity, regional homogeneity, independent component analysis, frequency domain, GT, or gradient analyses. The association of asymmetry and homotopy measures with the cerebellum, a region that has recently been associated with language, emotion, and social cognition, could encourage the use of these data in future studies.

## Data Availability Statement

The datasets generated for this study can be found in online repositories. The MRI dataset for this study can be found at https://openneuro.org/datasets/ds003481/. For the code analysis presented here, please check: https://jalilrt.github.io/Pragmatic-language-dataset-code/.

## Ethics Statement

The studies involving human participants were reviewed and approved by Committee on Ethics of Instituto de Neurobiología, Universidad Nacional Autónoma de México, which also approved the experimental protocol, in compliance with the federal guidelines of the Mexican Department of Health (http://www.salud.gob.mx/unidades/cdi/nom/compi/rlgsmis.html). The patients/participants provided their written informed consent to participate in this study.

## Author Contributions

JR-T conceived the idea. JR-T and VO-M performed the analysis. FL-C contributed to data and analysis checking. JR-T, FL-C, and AR-A verified quality control of the dataset, designed analysis, and wrote the paper. JR-T, FL-C, VO-M, GL-H, MZ-U, and AR-A collected the data. MG supervised the projects and contributed to the final version of the study. All authors contributed to the article and approved the submitted version.

## Conflict of Interest

The authors declare that the research was conducted in the absence of any commercial or financial relationships that could be construed as a potential conflict of interest.
