# Supplementary material for: A Dataset to Study Pragmatic Language and Its Underlying Cognitive Processes
Source: Front Hum Neurosci. 2021 Jun 17;15:666210. doi: 10.3389/fnhum.2021.666210 (PMC8248681; doi:10.3389/fnhum.2021.666210)
Supplement: Supplementary file 1 [file Data_Sheet_1.PDF]

Supplementary material

Whole descriptions on each task are in project json in the dataset.

Speech acts recognition task: In order to characterize the neural substrate of speech act recognition, three events categories were included, (Speech act, control YES and control NO events). Each trial in the fMRI session consisted of a sentence followed by a verb (that either named the speech act or was present or absent in the sentence). The sentence was displayed for 6000 ms, followed by a star (500 ms) and a verb (12000 ms). Events were separated by a fixation cross varying from 2000 to 12000 ms to prevent participants from anticipating the appearance of the next trial, and were presented randomly in a slow event related design. The fMRI session consisted of three runs; each run contained 20 events, 4 of which were SA events, 8 "control YES" and 8 "control NO". Each run lasted between 8.45 and 8.7 minutes. (Licea- Haquet et al, *in preparation*)

Speech acts - Emotion categorization task: In order to study how language comprehension is related to paralinguistics facial expression, we searched the relation between linguistically structured affirmative utterances and facial emotional expression (anger, sadness, happiness and a blurred neutral face). Participants were asked to sort of each utterance read according to if it was considered as a demand, earning request or an assertion. Each facial expression was referred to as a fMRI event with random presentation and random cross blank delay between each event. Whole task was divided into three runs. A four run, named Emotion - Face categorization task was designed to match the corresponding face expression with the word descriptor of it. Following the same presentation as the first run (Rasgado-Toledo et al, *in preparation*).

Proverb comprehension task: The aim of the study was to describe the cerebral correlates of the interpretation of Mexican proverbs using functional magnetic resonance imaging through an event-related paradigm. Participants were instructed to read an utterance that could have either with a literal meaning, a familiar proverb or a proverb with novelty meaning, after it, a second fixation cross appeared for a jittered time (6-10 seconds.), lastly, a second short utterance or a question was presented for 4 seconds, participants had to decide whether the second utterance carried the meaning of the first one, by pressing one of two buttons (yes/no). There were 26 trials in a run, that contained 4 familiar, 4 Novelty, and 18 Literal, in a completely randomized way, both between runs and subjects. There were 4 runs in the whole session (Carrillo-Peña, 2017, thesis).

Comprehension of metaphorical phrases: The aim of this research was to characterize the comprehension of metaphorical sentences with functional connectivity in a paradigm event-related functional Magnetic Resonance Imaging (fMRI). The experimental paradigm was divided in two runs. In each run we randomly presented 40 experimental events (20 literal phrases, 10 metaphorical phrases, 10 absurd phrases) on a black background within the MRI machine with the Psychopy program (Pierce, 2009). The experimental events were presented for 3500 ms, followed by a 5000 ms fixation cross, and the presentation for 3500 ms of a question in which they selected an answer ("YES" or "NO") with a pair of keypads compatible with the MRI. Each event had a separation interval by a fixation cross of 500 ms (Zamora-Ursulo, 2018, thesis).

Neuropsychological test-retest rsfMRI: Neuropsychological tests were applied to assess the consistency of different language abilities and resting state networks in a university student population within the span of 6 months. The acquisition of both the neuropsychological test and the resting state functional magnetic data was done in two moments: the first was at the beginning of the school semester and the second was 6 months later after the end of the semester. Of the initial participants (N= 30), only 16 completed the second assessment *(*Olalde-Mathieu et al.*, in preparation)*.

Lexical-semantic processing of verbs: In order to detect the difference in the recruitment of brain regions between pre-lexical and lexical-semantic processing, four stimuli categories were included (symbols, pseudo-verbs, motor-verbs, and mental-verbs) in a one-back detection task for an fMRI study. The stimuli were presented for 1000 ms with 2000 ms interstimulus intervals. Ten stimuli from one category were shown in each block, seven of the 10 stimuli were repeated randomly and subjects performed a one-back detection task. Blocks were separated by 12 sec blank intervals. Two blocks of each stimulus category were presented in each of eight runs. Duration per run was 6 min approximately (Reyes-Aguilar et al, *in preparation*).
